# Insights into implementation planning for point-of-care testing to guide treatment of chronic obstructive pulmonary disease exacerbation: a mixed methods feasibility study

**DOI:** 10.3389/frhs.2023.1302653

**Published:** 2024-01-03

**Authors:** Julie Hart, Alexander Daniel Edwards, Andrew Stainthorpe

**Affiliations:** ^1^ School of Pharmacy, University of Reading, Reading, United Kingdom; ^2^ Oxford University Hospitals NHS Foundation Trust, Oxford Academic Health Science Network, Oxford, United Kingdom; ^3^ Electronics and Computer Science, University of Southampton, Southampton, United Kingdom; ^4^ Research Health Limited, Corsham, United Kingdom

**Keywords:** insights, COPD exacerbation, implementation science, point-of-care testing (POCT), feasibility study, mixed methods, user-centered design, value-based pricing (VBP)

## Abstract

The purpose of this mixed methods feasibility study was to gain insights into unmet clinical needs, stakeholder preferences and potential barriers and enablers to adoption for planning the implementation of point-of-care testing for earlier detection and guided treatment of chronic obstructive pulmonary disease (COPD) acute exacerbation in the NHS in England. Exacerbations of COPD cause considerable mortality and morbidity. Earlier identification of exacerbations and guided treatment would lead to reduced exacerbation duration, reduced hospitalizations and mortality, improve health-related quality of life, reduce unnecessary treatments (including inappropriate antibiotic prescribing) which could save the NHS over £400 per patient. During the early stages of product design, we took a multi-disciplinary approach to evidence generation, gaining insights from key stakeholders to test the product concept and inform evidence-based implementation planning. Primary data was collected from 11 health care and service professionals involved in the management of acute COPD exacerbations. Overall, participants agreed that by earlier differentiation of acute exacerbation from stable COPD, patients could be started on appropriate treatment. To implement point-of-care testing into clinical practice, evidence is required to demonstrate the accuracy of differentiating between exacerbation etiologies and to provide information on the beneficial impact to the system in terms of optimized management, reduced long-term side effects, admission avoidance, and cost-effectiveness. This research provides an evidence base for future implementation planning of point-of-care testing for earlier detection and guided treatment of COPD acute exacerbation. Moreover, the technology developers can decide whether to refine the product design and value proposition thereby de-risking product development.

## 1. Introduction

Chronic obstructive pulmonary disease (COPD) is characterized by progressive airflow limitation. In 2019, over 200 million cases of COPD were reported leading to >3 million deaths globally (1). In the UK, 1.2 million people suffer from COPD costing health care and service providers >£800 million a year in treatment costs, and COPD is responsible for nearly 30,000 deaths annually (2). Exacerbation is the term used for acute and sustained worsening of COPD symptoms. COPD is irreversible but exacerbations are preventable by treatment and management with drugs such as steroids, beta_2_-agonists, antibiotics and vasodilators (3).

Acute exacerbations of COPD reduce the quality of life for patients, increase hospitalizations and are difficult to predict and detect early enough to intervene. Current strategies include the use of patient reported outcome measures (PROMs) and measurement of blood biomarkers or causative agents (4). PROMs have been combined into a smartphone-based algorithm with high diagnostic agreement (5). In the USA, remote respiratory rate monitoring has been proposed as an alternate strategy (6). Techniques to directly measure inflammation in the airways, such as bronchoscopy, bronchoalveolar lavage and biopsy, are too invasive for routine use (7). Systematic reviews recommend further investigation into measuring inflammatory biomarkers in blood, including fibrinogen (8), C-Reactive Protein (8, 9), IL-6 (8, 9) and TNF-alpha (9) to detect acute COPD exacerbation.

The need for more accurate, non-invasive analysis of lung inflammation has led to increasing interest in exhaled breath analysis and urinalysis as methods for identifying surrogates for airway inflammation (10). Use of fractional exhaled nitric oxide (FENO) in breath has been used to differentiate between asthma and COPD exacerbation alongside blood eosinophil counts (11). FENO can be used to guide appropriate therapy in a sub-set of COPD patients (12). However, one study shows that there may not be a link between FENO levels and COPD exacerbation suggesting that breath analysis, such as FENO, may not have clinical utility in identifying COPD patients experiencing acute exacerbation (13).

Use of urinalysis is well-evidenced and implemented in clinical practice for kidney disease and urinary tract infection (14). Measuring a panel of 10 inflammatory biomarkers in urine has been shown to differentiate between stable COPD and acute exacerbation (15). The aim of randomized control trial NCT04296318 was to establish if a point-of-care test measuring 10 inflammatory biomarkers in urine, alongside symptom monitoring, has utility in earlier identification of COPD acute exacerbation and differentiation from stable disease, with sufficient reliability. An early cost-utility analysis showed that measuring inflammatory biomarkers in urine to guide treatment of COPD patients experiencing an exacerbation may be highly cost-effective (16). Evidence exists of user acceptance of the approach (17). This feasibility study was embedded into NCT04296318 alongside a patient usability study led by Leicester, to gain insights from key stakeholders about the product concept and generate evidence to support future implementation planning.

Early economic evaluation of medical technologies helps to ensure that new interventions being implemented in care pathways are more likely to be accurate and cost-effective facilitating more rapid implementation (18). Allotty et al. describe the importance of “developing the critical evidence base that informs effective, sustained and embedded adoption of interventions by health systems and communities” (19). Key criteria for implementation of new technologies have been summarized into a checklist (20). Implementation is defined as “the processes or methods, techniques, activities, and resources that support the adoption, integration, and sustainment of evidence-based interventions into usual settings—sample indicators and outcomes include acceptability, adoption, appropriateness, cost, feasibility, penetration and sustainability” (21). Significant progress has been made developing frameworks to build and disseminate evidence that underpins implementation (21, 22) User-centered design can contribute greatly to evidence-based practice and driving successful implementation (23). The authors observe that test developers do not engage early enough with stakeholders who play a key role in influencing the implementation process. The purpose of this study was to gain insights, during the early stages of product design, into unmet clinical needs, stakeholder preferences and potential barriers and enablers to adoption to inform future development of an evidence-based implementation strategy for point-of-care testing for earlier detection and guided treatment of COPD acute exacerbation in the NHS in England.

## 2. Methods

In this feasibility study we took a multi-disciplinary approach to evidence generation, bringing together user-centered design, human factors, impact assessment and value-based pricing methods. Similar multi-disciplinary approaches have been developed, tested (24, 25) and supported early economic evaluation (26, 27). The purpose of this study was to gain insights from health care professionals (participants), working in hospitals and primary care, into the proposed implementation of point-of-care testing for earlier detection and guided treatment of acute exacerbation of COPD.

The study was conducted in accordance with the principles of the Declaration of Helsinki (2008) and the International Council for Harmonization and Good Clinical Practice guidelines and as part of NCT04296318 (COPE-WEL) approved by the Research Ethics Committee of University Hospitals of Leicester NHS Trust.

To identify stakeholders, a high-level care pathway for management of COPD acute exacerbation was mapped through discussions with service providers and by consulting the National Institute of Health and Care Excellence (NICE) guideline NG115 (28). Using a convenience sampling approach (29), participants were recruited from contacts already known to the researchers and from the participants' networks because it was essential that all participants were knowledgeable in the management of COPD patients and prescribing of appropriate therapies.

Recruitment was from March 2019 to June 2019. All participants provided informed consent for both the interview and recording of the interview. Participants completed a demographic questionnaire. A discussion guide was provided to participants describing the current care pathway and details of the proposed implementation of point-of-care testing.

(i) We used qualitative questions to capture participant's perspectives on the current care pathway:
  a. Definition of acute exacerbation
  b. Current methods for diagnosis and management
  c. Burden of inappropriate use of medicines
  d. Unmet need for an objective diagnostic test
  e. Long terms benefits from guided treatment
(ii) Participants were asked to rate their level of agreement on a 7-point Likert-type scale (30) against a series of questions to assess the utility of point-of-care testing in the care pathway where 1 = strongly disagree and 7 = strongly agree.
(iii) We used a standardized questionnaire to assess stakeholder preferences of perceived usefulness (31) where we asked participants to rate their level of agreement on a 7-point Likert-type scale.
(iv) Participants were asked about their intention to promote the use of point-of-care using a Net Promoter Score (32).
(v) To assess an acceptable price point, participants were presented with value-based scenarios and prompted to indicate the maximum price they would be willing to pay for point-of-care testing under the conditions in the scenario.
(vi) Participants were invited to consider factors that may influence their decision to adopt point-of-care testing for detection and guided treatment of acute exacerbation of COPD. They were provided with 5 key factors (cost and change to care pathway, patient outcomes, hospital admissions, prescribing) and asked to rate the level of impact these factors would have in the decision-making process (High/Medium/Low) and whether the impact would be positive or negative.
(vii) Participants were asked about the minimum level of sensitivity and specificity that would be acceptable.

Interviews lasted 30–45 min and were recorded using an audio recorder following verbal consent. No financial reimbursement was offered to participants. Each interview was manually transcribed and checked by another team member. The transcripts were analyzed and organized into themes. The saturation point was achieved where no new themes or opinions were observed. For the Likert-type items composite score and percentage level of agreement were calculated. The study output provides evidence for future implementation planning for point-of-care testing for earlier detection and guided treatment of COPD acute exacerbation.

## 3. Results

All participants had sufficient experience and were actively involved in the treatment of COPD patients. 36.4% of the participants interviewed were male with a mean of 19.8 years of experience and a mean age of 45.3. 63.6% of the participants interviewed were female with a mean of 13.9 years of experience and a mean age of 46.1 years.

### 3.1. Insights into the current care pathway and unmet clinical needs

We used qualitative questions to capture participant's perspectives on the current care pathway for COPD acute exacerbation and to assess the level of unmet need for an objective diagnostic test.

Q1. Participants defined an acute exacerbation of COPD as a sustained worsening of symptoms (increased breathlessness, increased sputum volume or production, sputum purulence, worsening cough and wheezing) beyond the patients' normal variation that required changes to their treatment. All participants concurred that the diagnosis of an acute exacerbation of COPD is currently based on symptoms and clinical assessment.
Q2. For the management of an acute exacerbation of COPD, treatment options cited were steroids, antibiotics, physiotherapy, beta_2_-agonists, bronchodilators, nebulized therapy, controlled oxygen, non-invasive ventilation and intubation. All participants acknowledged the vital role of the community care teams that are contacted by patients to conduct an initial assessment. Only 60% of the interviewees stated that patient self-management plans were well used in their region.
Q3. All participants agreed that there was a high level of inappropriate prescribing in the management of an acute exacerbation of COPD and all participants stated that this was not limited to antibiotics and steroids but existed across a range of treatment options.
Q4. All participants agreed that it may be useful to have an objective diagnostic test to direct towards appropriate treatments by differentiating exacerbation etiology, however the test would need to be used as an adjunct to symptom monitoring and clinical assessment.
Q5. All participants held the opinion that a reduction in steroid use would have long term benefits for patients and reduced antibiotic use would lead to wider population benefits.

We asked participants to rate their level of agreement to assess the utility of point-of-care testing in the COPD care pathway (Table 1).

**Table 1:** Participants’ level of agreement of the utility and need for implementing point-of-care testing for detection and guided treatment of acute exacerbation of COPD.

Questions	Level of agreement	Comments
Do you agree that there is an unmet need to change the care pathway for managing patients who are experiencing an acute exacerbation?	84%	Participants strongly felt that patients and non-specialist colleagues required more education on the management of an acute exacerbation of COPD.
Do you agree that that quicker patient recovery to the pre-exacerbation (baseline) state could be achieved by more appropriate and targeted treatment?	78%	Specialists in the field commented that there were increased risks of treatment failure if patients were given the incorrect treatment.
Do you agree that by targeting an infectious exacerbation with antibiotics only, this could eliminate unnecessary side effects from steroid use?	83%	By removing the use of oral steroids in these types of exacerbations, it was felt that the overall steroid burden on the patient would also be reduced, and this could result in downstream saving to the system from reduced side effects of long-term steroid use. However, participants emphasized that if an infection also brought on an inflammatory response, then steroids would be necessary.
Do you agree that targeted treatment with steroids for inflammatory exacerbations could eliminate unnecessary side effects?	84%	Treatment with steroids only would be beneficial to the patient but increasing antimicrobial resistance could negatively impact some cohorts of COPD patients.
Do you agree that appropriate and targeted treatment could potentially result in better patient compliance and adherence to medication?	71%	Participants stated that several other factors may also influence better patient compliance and adherence to medication, such as, improved health state and patient engagement in education and improved understanding of their condition.
Do you agree that the proposed test would be suitable for use by a healthcare professional either in clinic or when visiting the patient at home?	94%	If appropriate training was given and the test was easy to use. Two participants also proposed that there was scope to include other healthcare professionals.
Do you agree that this type of point-of-care test could be used by a patient as a self-test prior to using a rescue pack?	71%	Most participants remarked that this approach would only be suitable for a select cohort of patients that understood their condition, that patients followed the correct test procedure and that patients did not attempt to interpret the results themselves.
Do you agree that 10 min is an acceptable length of time to obtain the results from the test when conducted in a GP practice?	81%	Several participants were of the view that the pathway would need to be optimized and that by investing more time with the patient initially in conducting the test could avoid referrals and subsequent visits.
Do agree that connectivity to the electronic patient record would facilitate the adoption of this point-of-care test?	88%	Participants believed with robust record keeping of the test results, the test output could be used as a further education tool for patients and incorporated into their management plans and could be a more efficient way of monitoring patients’ disease and exacerbation events over time.
Do you agree that there are potential barriers in adoption for this point-of-care test?	75%	There would be significant barriers to adopting this test to differentiate COPD exacerbation etiology.
Do you agree that changes in the care pathway would be accepted for implementation of this point-of-care test?	79%	If the evidence supported guided therapeutic treatment prescribing based on differentiating COPD exacerbation etiology directed by a point-of-care test and was cost-effective.
Do you agree that this point-of-care test could help in improving the patient management by prescribing tailored and appropriate treatment?	91%	Participants acknowledged that it would need to be proven, however, they could foresee the potential benefits for individualized patient management.
Overall Average of agreement	82%	Reduction in unnecessary prescribing which was reflected in the overall level agreement.

### 3.2. Insights into stakeholder preferences

**Figure 1:**
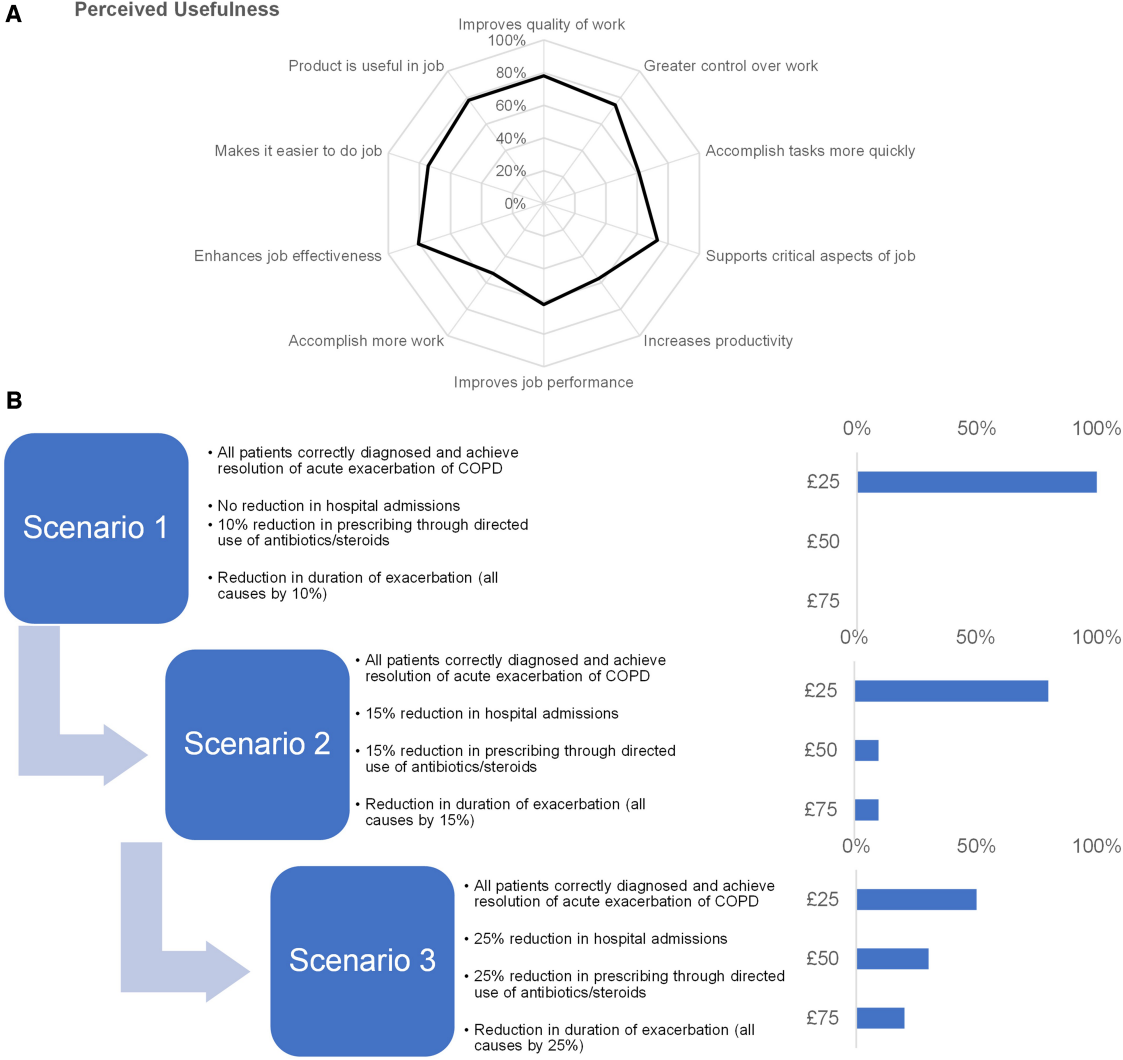
Insights into stakeholder preferences. (**A**) We used a standardized questionnaire to assess stakeholder preferences of perceived usefulness of point-of-care testing where we asked participants to rate their level of agreement on a 7-point Likert-type scale and plotted their responses on a spider chart. (**B**) To assess an acceptable price point, participants were presented with value-based scenarios (left) and prompted to indicate the maximum price they would be willing to pay for point-of-care testing under the conditions in the scenario (right).

We used a standardized questionnaire to summarize stakeholder preferences as to perceived usefulness (Figure 1A). All participants appreciated the clinical utility of determining treatment options in the event of acute exacerbation. However, evidence would be required to determine the utility of the inflammatory biomarkers in the differentiation of COPD exacerbation etiology and that point-of-care testing for detection and guided treatment of acute exacerbation of COPD was cost-effective.

Participants were asked about their intention to promote the use of point-of-care using a Net Promoter Score. 73% of participants identified themselves as a potential promoter for using point-of-care testing citing the test results had the potential to translate into actionable treatments. The remaining 27% participants were neutral due to lack of studies to show technical performance, clinical validity and system and patient benefits. There were no detractors.

We asked participants to assess an acceptable price point when presented with value-based scenarios (Figure 1B). For scenario 1, all participants agreed that price point of £25 was acceptable. 80% of participants also selected the £25 price point for scenario 2, with the remainder of participants split between the £50 and £75 price point. For scenario 3, 50% of the participants would accept a higher price point with 30% of participants accepting £50% and 20% participants accepting £75. The remaining 50% of participants cited the maximum acceptable price as £25.

### 3.3. Insights into key decision factors

For each key decision factor an impact assessment was made based on participant ratings.

Factor 1: Cost was identified as a major potential barrier with evidence required to show benefits in terms of optimized medical management, reduced long-term side effects and cost-effectiveness. All participants rated the cost of introducing point-of-care testing as having high and negative impact.
Factor 2: 45% of participants said that changing the care pathway would have high impact, 55% said it would have medium impact. 91% of participants felt that changes would have positive impact. Further information would need to be provided regarding the specific patient cohorts suitable for point-of-care testing.
Factor 3: All participants felt that the changes to patient outcomes would have high and positive impact and further evidence from clinical validation was needed to demonstrate improvement in patient outcomes.
Factor 4: 82% of participants believed that the potential reductions in hospital admissions would have high impact, with all stakeholders indicating that this would have positive impact as it should translate into cost-savings and service efficiencies.
Factor 5: 91% of participants commented that changes to prescribing would have high impact, with 100% of participants concurring that this would have positive impact. A proven reduction in overprescribing would impact acceptance of point-of-care testing especially if there was potential to prevent the patient from deteriorating by using point-of-care testing.
Factor 6: An average of 84% for sensitivity and 85% for specificity was calculated as acceptable performance levels but robust evidence was required to demonstrate that point-of-care testing accurately differentiated between exacerbation types.

Specific participant quotes regarding key decision factors included:

• *Respiratory clinician: “Cost. It needs to be in the system where it is financially viable to have it implemented”*
• *Pharmacist: “Patients with COPD have other comorbidities, there would need to be information and evidence regarding confounding factors”*
• *Respiratory clinician: “Evidence for accuracy from a clinical validation is required to overcome concerns around false positives and false negatives”*
• *Specialist respiratory nurse: “Need evidence for benefits to the system and reduced/appropriate use of medications”*

Specific participant quotes regarding the acceptance in changing the care pathway to incorporate point-of-care testing included:

• *Pharmacist: “We already phenotype patients, so it would be accepted here”*
• *Specialist respiratory nurse: “Treatment has been stagnated for a long time… we are not doing it correctly and there are different trajectories depending on the biological mechanism”*
• *GP: “If it's proven to be cost-effective and makes a difference, we will use it”*

## 4. Discussion

From our interviews we gained an understanding of the key decision factors regarding adoption and implementation of point-of-care testing for earlier detection and guided treatment of COPD acute exacerbation. The evidence we have generated can be be used in developing an implementation strategy. There was agreement that this point-of-care test for earlier detection and guided treatment of acute exacerbation of COPD could be used constructively. Overall, participants agreed that by earlier differentiation of acute exacerbation from stable COPD, patients could be started on the correct treatment (particularly by non-specialists) and use of targeted therapies could lead to a reduction in the use of steroids and inappropriate use of antibiotics.

Next steps for implementation of this point-of-care test includes defining the optimal point for use in the care pathway driven by the key decision-making factors noting that participants raised concerns regarding patients interpreting tests themselves without professional input. Key decision factors are cost and performance (test sensitivity and specificity above 85%). We have identified that to implement the test in clinical practice, more evidence would be required to demonstrate the accuracy of differentiating between exacerbation etiologies and provide evidence on the beneficial impact in terms of optimized management, improvements in patient outcomes, reduced long-term steroid-burden side effects, lower rates of hospitalizations, steroid and antibiotic use, overall cost reduction and cost-effectiveness. A key opportunity was identified in that there was support for funding a point-of-care test which offered accurate diagnosis at £25 per test, and up to £75 per test for incremental reductions in exacerbation rates and hospitalizations.

The output from mixed methods feasibility studies (24) and multi-dimensional processes (25) can be incorporated into Target Product Profiles to support the design of “fit for purpose” medical technologies (33). To our knowledge, this is the first study to embed a mixed methods feasibility study measuring participant's acceptance of point-of-care testing, into a randomized control trial to generate evidence to guide implementation planning in the COPD care pathway.

In this feasibility study, both qualitative and quantitative methods are used. Interview protocols for qualitative research can deliver a robust evidence base (34) and can be incorporated within a mixed methods approach (35). For analysis of Likert scale data, Norman supports the use of parametric tests (36) but conclusions are similar using parametric or non-parametric tests (37). Care needs to be taken not to misuse Likert scales (38) and the composite score can be calculated without using a statistical test (39). Such approaches are useful where the researcher is investigating the prevalence of behaviors and preferences of participants and wants to connect the data in a single unified result.

The authors recognize the wide range of tools and methodologies available for the early assessment of innovative medical devices including diagnostics. Horizon-scanning helps policy makers to understand the innovation landscape to guide policy development (40), and Multi Criteria Decision Analysis can support regulatory agencies in health technology assessment and priority setting (41). Early technology assessment and early economic evaluation helps innovators to align their products with the specific needs of the market and supports commercialization (42). There is a growing interest across health care and early health technology assessment is gaining in momentum (43). A systematic review identified −1,200 references for value assessment in health care innovation between 2007 and 2017 with 38 methodologies and frameworks identified (44).

Responsible Research and Innovation (RRI) emphasizes the importance of ensuring that publicly funded research and innovation is aligned with unmet needs, stakeholder views and iterative design towards commercialization. RRI recommends use of a development framework across value domains in the product development lifecycle (45). We propose that a mixed methods feasibility study adds value in an iterative product design process and supports the development of implementation strategies, alongside patient and public involvement. Frameworks including non-adoption, abandonment, scale-up, spread and sustainability (NASSS) considers challenges beyond the initial implementation phase (46) as demonstrated within the field of cardiovascular medicine (47). This mixed methods feasibility study tested an early-stage diagnostic product. We have successfully used this approach to generate evidence to guide implementation planning for a market ready test (48, 49). Future research should further explore this approach across different technology areas and expanded geographical coverage. Moreover, NASSS could provide a comprehensive framework for guiding future feasibility studies to support early evidence generation to drive adoption, implementation, scale and spread.

## Data Availability

The datasets presented in this article are not readily available because of restrictions in legal agreements related to data sharing. Requests to access the datasets should be directed to julie.hart@reading.ac.uk.

## References

[1] Safiri S Carson-Chahhoud K Noori M Nejadghaderi SA Sullman MJM Ahmadian Heris J Burden of chronic obstructive pulmonary disease and its attributable risk factors in 204 countries and territories, 1990–2019: results from the global burden of disease study 2019. Br Med J. (2022) 378: e069679. 10.1136/bmj-2021-069679 35896191 PMC9326843

[2] Wright A Vioix H de Silva S Langham S Cook J Capstick T Cost-consequence analysis of COPD treatment according to NICE and GOLD recommendations compared with current clinical practice in the UK. BMJ Open. (2022) 12(6):e059158. 10.1136/bmjopen-2021-059158 36691251 PMC9171279

[3] Agustí A Celli BR Criner GJ Halpin D Anzueto A Barnes P Global initiative for chronic obstructive lung disease 2023 report: GOLD executive summary. Eur Respir J. (2023) 61(4):2300239. 10.1183/13993003.00239-2023 36858443 PMC10066569

[4] Chen Y Zhang J Curtis JL. Editorial: toolkits for prediction and early detection of acute exacerbations of chronic obstructive pulmonary disease. Front Med (Lausanne). (2022) 9:899450. 10.3389/fmed.2022.899450 35573020 PMC9093642

[5] Claxton S Porter P Brisbane J Bear N Wood J Peltonen V Identifying acute exacerbations of chronic obstructive pulmonary disease using patient-reported symptoms and cough feature analysis. NPJ Digit Med. (2021) 4(1):107. 10.1038/s41746-021-00472-x 34215828 PMC8253790

[6] Polsky MB Moraveji N. Early identification and treatment of COPD exacerbation using remote respiratory monitoring. Respir Med Case Rep. (2021) 34:101475. 10.1016/j.rmcr.2021.101475 34367906 PMC8326429

[7] Afroz N Gutzwiller FS Mackay AJ Naujoks C Patalano F Kostikas K. Patient-reported outcomes (PROs) in COPD clinical trials: trends and gaps. Int J Chron Obstruct Pulmon Dis. (2020) 15:1789–800. 10.2147/COPD.S235845 32801678 PMC7398869

[8] Fermont JM Masconi KL Jensen MT Ferrari R Di Lorenzo VAP Marott JM Biomarkers and clinical outcomes in COPD: a systematic review and meta-analysis. Thorax. (2019) 74(5):439–46. 10.1136/thoraxjnl-2018-211855 30617161 PMC6484697

[9] Chen YW Leung JM Sin DD. A systematic review of diagnostic biomarkers of COPD exacerbation. PLoS One. (2016) 11(7):e0158843. 10.1371/journal.pone.0158843 27434033 PMC4951145

[10] van Velzen P Brinkman P Knobel HH van den Berg JWK Jonkers RE Loijmans RJ Exhaled breath profiles before, during and after exacerbation of COPD: a prospective follow-up study. COPD. (2019) 16(5-6):330–7. 10.1080/15412555.2019.1669550 31588813

[11] Katoh S Ikeda M Shirai R Abe M Ohue Y Kobashi Y Biomarkers for differentiation of patients with asthma and chronic obstructive pulmonary disease. J Asthma. (2018) 55(10):1052–8. 10.1080/02770903.2017.1391281 29035604

[12] Su KC Ko HK Hsiao YH Chou KT Chen YW Yu WK Fractional exhaled nitric oxide guided-therapy in chronic obstructive pulmonary disease: a stratified, randomized, controlled trial. Arch Bronconeumol. (2022) 58(8):601–10. 10.1016/j.arbres.2021.11.013 35312525

[13] Lu Z Huang W Wang L Xu N Ding Q Cao C. Exhaled nitric oxide in patients with chronic obstructive pulmonary disease: a systematic review and meta-analysis. Int J Chron Obstruct Pulmon Dis. (2018) 13:2695–705. 10.2147/COPD.S165780 30214187 PMC6124452

[14] Simerville JA Maxted WC Pahira JJ. Urinalysis: a comprehensive review. Am Fam Physician. (2005) 71(6):1153–62. PMID: 15791892 15791892

[15] Yousuf AJ Parekh G Duvoix A Parker S Finch J Glover S Changes in urinary biomarkers between stable state and exacerbation of COPD. Eur Resp J. (2018) 52(suppl 62):PA4069. 10.1183/13993003.congress-2018.PA4069

[16] Abel L Dakin HA Roberts N Ashdown HF Butler CC Hayward G Is stratification testing for treatment of chronic obstructive pulmonary disease exacerbations cost-effective in primary care? An early cost-utility analysis. Int J Technol Assess Health Care. (2019) 35(2):116–25. 10.1017/S0266462318003707 30829566

[17] Stainthorpe A Hart J Bajre M. Novel methodology developed to assess feasibility of new urine-based biomarker test for respiratory illness. Value Health. (2019) 22:S881. 10.1016/j.jval.2019.09.2541

[18] Smith AF Sutton A Shinkins B. Early cost-effectiveness analysis of new medical tests: response. Int J Technol Assess Health Care. (2016) 32(4):324–5. 10.1017/S026646231600043X 27691992

[19] Allotey P Reidpath DD Ghalib H Pagnoni F Skelly WC. Efficacious, effective, and embedded interventions: implementation research in infectious disease control. BMC Public Health. (2008) 8:343. 10.1186/1471-2458-8-343 18826655 PMC2567977

[20] Guldbrandsson K. From news to everyday use: The difficult art of implementation. Statens Folkhälsoinstitut, Rapport R (2008) (9). https://www.folkhalsomyndigheten.se/publikationer-och-material/publikationsarkiv/f/from-news-to-everyday-use.-the-difficult-art-of-implementation

[21] Brownson RC Shelton RC Geng EH Glasgow RE. Revisiting concepts of evidence in implementation science. Implement Sci. (2022) 17(1):26. 10.1186/s13012-022-01201-y 35413917 PMC9004065

[22] Treweek S Oxman AD Alderson P Bossuyt PM Brandt L Brożek J Developing and evaluating communication strategies to support informed decisions and practice based on evidence (DECIDE): protocol and preliminary results. Implement Sci. (2013) 8(6). 10.1186/1748-5908-8-6 PMC3553065 23302501

[23] Dopp AR Narcisse MR Mundey P Silovsky JF Smith AB Mandell D A scoping review of strategies for financing the implementation of evidence-based practices in behavioral health systems: state of the literature and future directions. Implement Res Pract. (2020) 1:2633489520939980. 10.1177/2633489520939980 37089129 PMC9924261

[24] Ni M Borsci S Bajre M Buckle P Oh S Heitmueller A The lean assessment process (LAP) for developing early-stage medical technologies—experiences of NIHR London IVD cooperative. SS-09 guidelines on the early assessment of biomedical innovations using multiple criteria. IUPESM Prague 2018 World Congress on Medical Physics and Biomedical Engineering.

[25] Ni M Borsci S Walne S Mclister AP Buckle P Barlow JG The lean and Agile multi-dimensional process (LAMP)—a new framework for rapid and iterative evidence generation to support health-care technology design and development. Expert Rev Med Devices. (2020) 17(4):277–88. 10.1080/17434440.2020.1743174 32167800

[26] Bajre M Moawad M Shumbayawonda E Carolan JE Hart J Culver E Livermultiscan as an alternative to liver biopsy to monitor autoimmune hepatitis in the national health service in England: an economic evaluation. BMJ Open. (2022) 12(9):e058999. 10.1136/bmjopen-2021-058999 36691214 PMC9462097

[27] Bajre M Hart J Stainthorpe A. High sensitivity troponin point of care testing for cost-effective early rule out of acute myocardial infarction within the emergency department: an early economic evaluation. Value Health. (2020) 23:S494. 10.1016/j.jval.2020.08.534

[28] National Institute for Health and Care Excellence. *Chronic obstructive pulmonary disease in over 16s*. (2019). Available at: https://www.nice.org.uk/guidance/ng115 (Accessed: February 09, 2019).

[29] Stratton SJ. Population research: convenience sampling strategies. Prehosp Disaster Med. (2021) 36(4):373–4. 10.1017/S1049023X21000649 34284835

[30] Likert R. A technique for the measurement of attitudes. Arch Psychol. (1932) 140:1–55.

[31] Davis FD. User acceptance of information technology: system characteristics, user perceptions and behavioral impacts. Int J Man Mach Stud. (1993) 38(3):475–87. 10.1006/imms.1993.1022

[32] Reichheld FF. One number you need to grow. Harv Bus Rev. (2003) 1(12):46–54, 124. 14712543

[33] Cocco P Ayaz-Shah A Messenger MP West RM Shinkins B. Target product profiles for medical tests: a systematic review of current methods. BMC Med. (2020) 18(1):119. 10.1186/s12916-020-01582-1 32389127 PMC7212678

[34] Busetto L Wick W Gumbinger C. How to use and assess qualitative research methods. Neurol Res Pract. (2020) 2:14. 10.1186/s42466-020-00059-z 33324920 PMC7650082

[35] Guest G Fleming PJ. Mixed methods research. In: Guest G Namey E, editors. Public health research methods. Thousand Oaks, CA: Sage (2014).

[36] Norman G. Likert scales, levels of measurement and the “laws” of statistics. Adv Health Sci Educ Theory Pract. (2010) 15(5):625–32. 10.1007/s10459-010-9222-y 20146096

[37] Murray J. Likert Data: what to use, parametric or non-parametric? Int J Bus Soc Sci. (2013) 4:258–64.

[38] Carifio J Perla RJ. Ten common misunderstandings, misconceptions, persistent myths and urban legends about Likert scales and Likert response formats and their antidotes. J Soc Sci. (2007) 3(3):106–16. 10.3844/jssp.2007.106.116

[39] Carifio J Perla R. Resolving the 50-year debate around using and misusing Likert scales. Med Educ. (2008) 42(12):1150–2. 10.1111/j.1365-2923.2008.03172.x 19120943

[40] Vignali V Hines PA Cruz AG Ziętek B Herold R. Health horizons: future trends and technologies from the European medicines agency’s horizon scanning collaborations. Front Med (Lausanne). (2022) 9:1064003. 10.3389/fmed.2022.1064003 36569125 PMC9772004

[41] Baltussen R Marsh K Thokala P Diaby V Castro H Cleemput I Multicriteria decision analysis to support health technology assessment agencies: benefits, limitations, and the way forward. Value Health. (2019) 22(11):1283–8. 10.1016/j.jval.2019.06.014 31708065

[42] Wang Y Rattanavipapong W Teerawattananon Y. Using health technology assessment to set priority, inform target product profiles, and design clinical study for health innovation. Technol Forecast Soc Change. (2021) 172:121000. 10.1016/j.techfore.2021.121000 34732945 PMC8524319

[43] Ijzerman MJ Steuten LM. Early assessment of medical technologies to inform product development and market access: a review of methods and applications. Appl Health Econ Health Policy. (2011) 9(5):331–47. 10.2165/11593380-000000000-00000 21875163

[44] Seixas BV Dionne F Conte T Mitton C. Assessing value in health care: using an interpretive classification system to understand existing practices based on a systematic review. BMC Health Serv Res. (2019) 19(1):560. 10.1186/s12913-019-4405-6 31409369 PMC6693163

[45] Pacifico Silva H Lehoux P Miller FA Denis JL. Introducing responsible innovation in health: a policy-oriented framework. Health Res Policy Syst. (2018) 16(1):90. 10.1186/s12961-018-0362-5 30200985 PMC6131953

[46] Greenhalgh T Wherton J Papoutsi C Lynch J Hughes G A'Court C Beyond adoption: a new framework for theorizing and evaluating nonadoption, abandonment, and challenges to the scale-up, spread, and sustainability of health and care technologies. J Med Internet Res. (2017) 19(11):e367. 10.2196/jmir.8775 29092808 PMC5688245

[47] Winter PD Chico TJA. Using the non-adoption, abandonment, scale-up, spread, and sustainability (NASSS) framework to identify barriers and facilitators for the implementation of digital twins in cardiovascular medicine. Sensors (Basel). (2023) 23(14):6333. 10.3390/s23146333 37514627 PMC10385429

[48] Hart J Checketts G. *PLGF-based testing to help diagnose suspected pre-eclampsia, PlGF-based testing to help diagnose suspected pre-eclampsia. Implementation support pack for provider organisations*. (2019). Available at: https://www.healthinnovationoxford.org/wp-content/uploads/2019/10/Implementation_pack_PIGF_test_pre-eclampsia.pdf (Accessed: November 28, 2023).

[49] Checketts G. *Two-thirds of maternity units in England adopt test to rule out pre-eclampsia following roll-out led by Oxford AHSN. Oxford AHSN case study*. (2021). Available at: https://www.healthinnovationoxford.org/wp-content/uploads/2021/05/Q4-Case-Study-PlGF-with-logo.pdf (Accessed: November 28, 2023).

